# Sterol Metabolism and Transport in Atherosclerosis and Cancer

**DOI:** 10.3389/fendo.2018.00509

**Published:** 2018-09-19

**Authors:** Yoshio Yamauchi, Maximillian A. Rogers

**Affiliations:** ^1^Nutri-Life Science Laboratory, Department of Applied Biological Chemistry, Graduate School of Agricultural and Life Sciences, University of Tokyo, Tokyo, Japan; ^2^AMED-CREST, Japan Agency for Medical Research and Development, Tokyo, Japan; ^3^Division of Cardiovascular Medicine, Center for Interdisciplinary Cardiovascular Sciences, Brigham and Women's Hospital, Harvard Medical School, Boston, MA, United States

**Keywords:** ABC transporters, cholesterol, lanosterol, oxysterols, cholesterol efflux, intracellular cholesterol transport, atherosclerosis, cancer

## Abstract

Cholesterol is a vital lipid molecule for mammalian cells, regulating fluidity of biological membranes, and serving as an essential constituent of lipid rafts. Mammalian cells acquire cholesterol from extracellular lipoproteins and from *de novo* synthesis. Cholesterol biosynthesis generates various precursor sterols. Cholesterol undergoes metabolic conversion into oxygenated sterols (oxysterols), bile acids, and steroid hormones. Cholesterol intermediates and metabolites have diverse and important cellular functions. A network of molecular machineries including transcription factors, protein modifiers, sterol transporters/carriers, and sterol sensors regulate sterol homeostasis in mammalian cells and tissues. Dysfunction in metabolism and transport of cholesterol, sterol intermediates, and oxysterols occurs in various pathophysiological settings such as atherosclerosis, cancers, and neurodegenerative diseases. Here we review the cholesterol, intermediate sterol, and oxysterol regulatory mechanisms and intracellular transport machineries, and discuss the roles of sterols and sterol metabolism in human diseases.

## Introduction

Sterol biosynthesis is thought to have evolved 2.31 billion years ago (1). In eukaryotes, various complex sterols exist among plants, yeast, and mammals, and a few bacteria are also capable of synthesizing simpler sterols. Cholesterol is a vital lipid molecule for all mammals, and plays diverse and important roles in a number of biological processes, physiology, and disease (2). Mammalian cells can acquire cholesterol from two sources; uptake from extracellular milieu (exogenous source) and *de novo* synthesis (endogenous source). In addition to cholesterol, cells produce a variety of sterols. In the process of cholesterol synthesis, a series of intermediate sterols are generated as cholesterol precursors. Cholesterol is metabolized to cholesteryl ester (CE), oxysterols, bile acids, and steroid hormones. All these non-cholesterol sterols have important physiological functions in cells and in tissues.

Cholesterol is an important constituent of cellular membranes, regulating membrane fluidity and functionality. Cellular cholesterol distribution is highly heterogeneous among organelles [reviewed in (3–5)]. The plasma membrane (PM) contains 60–90% of total cellular cholesterol, which accounts for 25–40 mol% of PM lipids. The endocytic compartments and *trans*-Golgi network (TGN) are also cholesterol-rich organelles; whereas, the endoplasmic reticulum (ER) and the mitochondria contain only ~1% of total cellular cholesterol, which accounts for ~5 mol% of ER lipids. Cholesterol is an essential constituent of membrane domains known as lipid rafts, which are small domains enriched in cholesterol and sphingolipids [both sphingomyelin (SM) and glycosphingolipids] [reviewed in (6)]. Lipid rafts are involved in key cellular functions like endocytosis, cellular signaling, and cell motility (6). In cells, cholesterol and other sterol molecules move dynamically among organelles to maintain proper distribution.

Aberrant accumulation of unesterified cholesterol in membranes is toxic to cells; therefore, various intrinsic and elaborate systems cooperatively regulate cellular sterol homeostasis. Cellular cholesterol content is tightly controlled by regulating *de novo* synthesis, extracellular uptake, export to extracellular milieu, and metabolic conversion (7, 8). Impairments in sterol homeostasis can cause various congenital and acquired diseases in humans, and pathophysiological conditions can also affect sterol homeostasis (2).

In this review, we provide an up-to-date assessment of the cell-intrinsic regulatory mechanisms for biosynthesis, intracellular transport, and efflux of cholesterol, intermediate sterols, and oxysterols. Additionally, we describe the roles of these sterol molecules in human diseases. This review contains four broad topics; (1) sterol biosynthesis and regulation, (2) intracellular sterol transport, (3) cellular sterol export, and (4) the roles of sterols in human diseases. Finally, we highlight several areas of research where mechanistic clarification is needed for sterol-related disease therapeutic development.

## Roles of sterols

Cholesterol, intermediate sterols, and oxysterols have various important functions; Table 1 summarizes some of the functions further described in this review.

**Table 1:** Roles of intermediate sterols, cholesterol, and oxysterols.

**Sterols**	**Biological activities**	**References**
Lanosterol	Inhibition of lens protein aggregation	(9)
Dihydrolanosterol	Stimulation of HMGCR ubiquitination	(10, 11)
FF-MAS	Meiosis activation	(12)
T-MAS	Meiosis activation	(12)
Desmosterol	Binding and regulation of SCAP, LXR ligand	(13, 14)
Cholesterol	Lipid raft formation	(6)
	Binding and regulation of SCAP	(13)
	Modification of Hedgehogs and Smoothend	(15, 16)
7α-OHC	Major bile acid precursor	(17)
24(S)-OHC	INSIG ligand, LXR ligand	(18, 19)
25-OHC	INSIG ligand, LXR ligand, anti-viral effect	(18–20)
27-OHC	INSIG ligand, LXR ligand, SERM	(18, 19, 21)
7α,25-di-OHC	EBI2 ligand	(22, 23)

### Role of cholesterol

Cholesterol is a membrane lipid that is indispensable for integrity of biological membranes. It is vital for forming lipid rafts, membrane nano-domains enriched in cholesterol, and sphingolipids, which play a variety of important roles in mammalian cells (6). Cholesterol can regulate functions of biological membranes including endocytosis, membrane trafficking, and signaling. Cholesterol is the precursor of steroid hormones, bile acids, and oxysterols. In addition, cholesterol modifies select proteins: it is covalently attached to Hedgehog proteins (15) and to smoothened (16), both of which work in concert in Hedgehog singling, a signaling pathway playing a critical role in embryonic development and tumorigenesis. As described in more detail below, cholesterol partly regulates cholesterol biosynthesis.

### Role of intermediate sterols

Sterol intermediates are not just precursors of cholesterol but can also act as biologically active agents. Lanosterol, the first biosynthesized sterol in the cholesterol biosynthetic pathway, can prevent lens protein aggregation and cataracts (9). Lanosterol treatment partially corrects cataracts in animal models. Zhao et al. (9) demonstrated that missense mutations in the lanosterol synthase *LSS* gene cause congenital cataracts. Dihydrolanosterol promotes ubiquitination and degradation of 3-hydroxy-3-methylglutaryl (HMG)-CoA reductase (HMGCR), inactivating cholesterol biosynthesis rapidly (10, 11). 4,4-dimethylcholesta-8,14,24-trien-3β-ol (follicular fluid meiosis-activating sterol, FF-MAS) and 4,4-dimethylcholesta-8,24-dien-3β-ol (testis-MAS, T-MAS) are implicated as meiosis-activating substances in oocyte maturation (12). In addition to cholesterol as described in the following section, desmosterol binds to sterol regulatory element binding protein (SREBP) cleavage activating protein (SCAP), and blocks SREBP activation to regulate cholesterol homeostasis (13). Desmosterol is also known to act as an endogenous ligand for the nuclear receptor liver X receptor (LXR) (14). Defects in enzymes involved in conversion of lanosterol to cholesterol cause severe malformation observed in Smith-Lemli-Optiz syndrome, desmosterolosis, X-linked dominant chondrodysplasia punctate type 2 (CDPX2), CHILD syndrome, Greenberg dysplasia, and Antley-Bixler syndrome (24, 25). In these patients, intermediate sterols accumulate in tissues and plasma.

### Role of oxysterols

Oxysterols are one of the most potent negative regulators of cholesterol biosynthesis. Side-chain oxysterols including 25-hydroxycholesterol (25-OHC), 27-OHC, and 24(*S*)-OHC bind INSIG proteins (18), which are ER-resident proteins involved in negative feedback regulation of cholesterol homeostasis. Recent work established virus infection activates conversion of cholesterol to 25-OHC, which inhibits the SREBP pathway and down-regulates cholesterol biosynthesis (20). 25-OHC formation-dependent suppression of cholesterol synthesis plays an important role in viral infection and replication inhibition. In addition, oxysterols act as LXR ligands (19) that induce expression of genes involved in cholesterol efflux, including ATP-binding cassette (ABC) transporters ABCA1 and ABCG1, and apolipoprotein E (apoE) [reviewed in (26)]. Furthermore, recent studies demonstrated that 27-OHC can bind to estrogen receptors and act as an endogenous selective estrogen receptor modulator (SERM) (21). Side-chain oxysterols, but not sterol ring modified oxysterols, show membrane-disordering effects that result in alternations in membrane functions (27).

## Sterol synthesis and its regulation

### Biosynthesis of intermediate sterols and cholesterol

In mammals, virtually all cells are capable of synthesizing cholesterol *de novo*. In humans, the liver is the most active organ in cholesterol synthesis, synthesizing as much as 1 g of cholesterol per day. Biosynthesis of cholesterol from the simple, two-carbon acetyl-CoA is a complex, multi-step process involving over 30 enzymes, and consumes 18 ATP a cholesterol molecule. Cholesterol biosynthesis begins with condensation of acetyl-CoA and acetoacetyl-CoA, which results in formation of HMG-CoA (28). A key rate-limiting enzyme in cholesterol biosynthesis is the ER membrane-bound enzyme HMGCR, which catalyzes reduction of HMG-CoA to mevalonate. This reaction uses NADPH as the reducing agent. Mevalonate is then phosphorylated by mevalonate kinase and phosphomevalonate kinase to yield 5-pyrophosphomevalonate. Subsequent isopentenyl pyrophosphate (IPP) production, a five-carbon (C5) isoprene unit, serves as precursor for all isoprenoids. IPP undergoes isomerization to 3,3-dimethylallyl pyrophosphate (DPP). DPP then condenses with IPP, yielding C10 geranyl pyrophosphate (GPP). Condensation of GPP and IPP produces C15 farnesyl pyrophosphate (FPP). Two molecules of FPP are then condensed and reduced to yield C30 squalene, the final non-sterol precursor of cholesterol. Squalene synthase catalyzes this reacting using NADPH as the reducing agent. Oxidation and cyclization of squalene yield C30 lanosterol, the first sterol in the biosynthetic pathway, via 2,3-epoxysqualene; squalene monooxygenase (also known as squalene epoxidase) that converts squalene to 2,3-epoxysqualene using NADPH and molecular oxygen, and lanosterol synthase then catalyzes the cyclization of 2,3-epoxysqualene to lanosterol.

**Figure 1:**
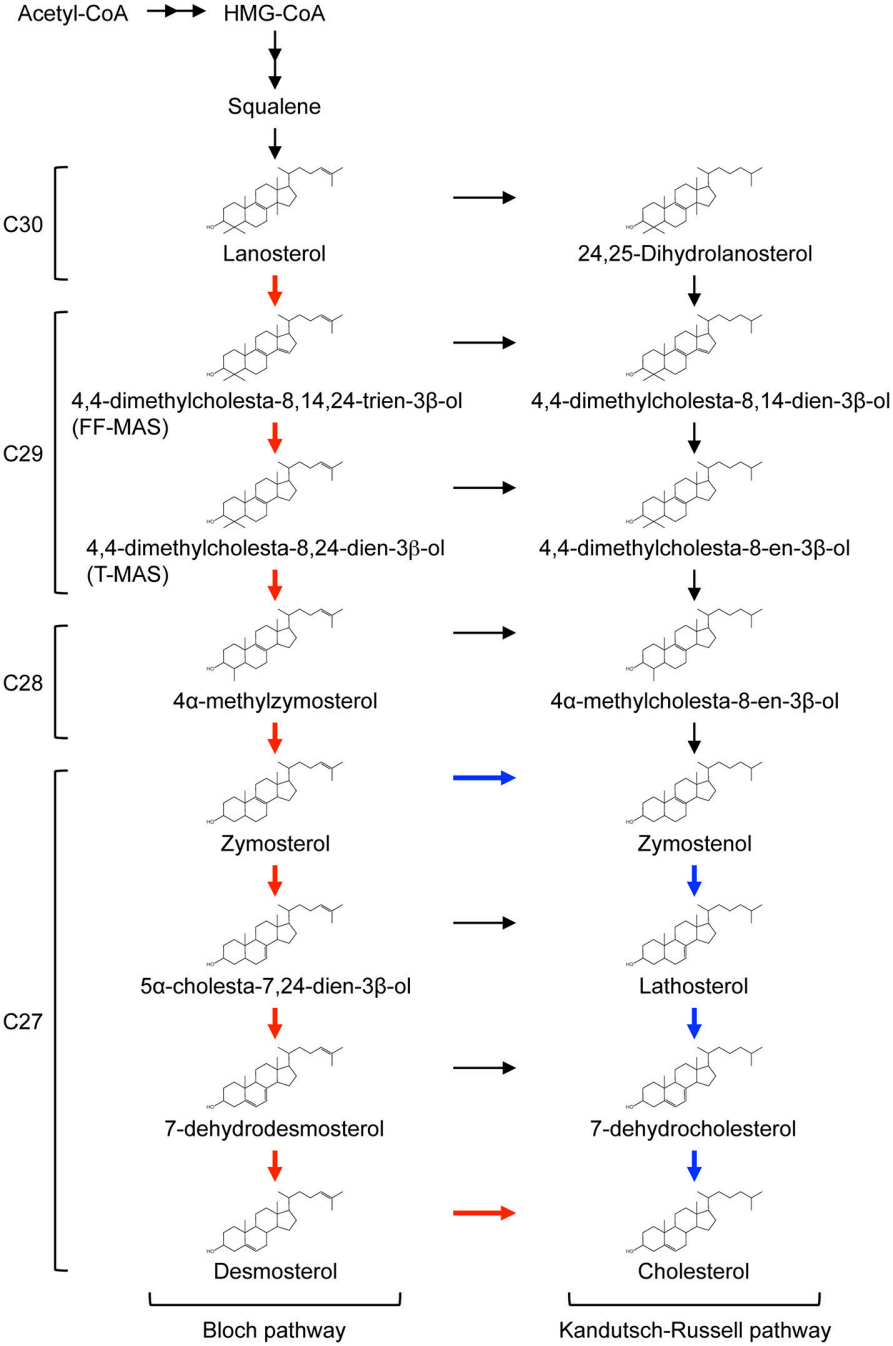
Cholesterol biosynthetic pathway.The conversion of lanosterol to cholesterol proceeds through either Bloch pathway (red line) or Kandutsch-Russell pathway. Recently, modified Kandutsch-Russell pathway (blue line) has been proposed (29). See text for more details. The number of carbons in each sterol is presented on the left of the pathway.

Lanosterol undergoes extensive modifications en route to the final product, cholesterol (Figure 1). Conversion of lanosterol to C27 cholesterol involves at least 18 different enzymatic reactions including removal of the three methyl groups, reduction of the side chain, and rearrangement of the double bonds within sterol rings by consuming NADPH and O_2_. All of the enzymes responsible for converting squalene to cholesterol localize in the ER membrane. Conversion of lanosterol to cholesterol proceeds through either one of two pathways known as Bloch pathway and Kandutsch-Russell pathway (Figure 1). Desmosterol and 7-dehydrocholesterol are the final precursors in the Bloch pathway and the Kandutsch-Russell pathway, respectively. The sterol Δ24-reductase, DHCR24, which catalyzes reduction of the side chain at position 24 using NADPH as the reducing agent, is a key branching enzyme for the two pathways. 14α-methyl group of lanosterol and dihydrolanosterol is first removed by lanosterol 14α-demethylase (CYP51A1), yielding C29 sterols with two methyl groups at C4 position. The two methyl groups are then sequentially trimmed by a series of complex reactions involving three enzymes, methylsterol monooxygenase 1 (encoded by *MSMO1*/*SC4MOL*), sterol-4α-carboxylate 3-dehydrogenase (NSDHL), and 3-keto-steroid reductase (HSD17B7), producing C27 zymosterol or zymostenol. Conversion of zymosterol or zymostenol to cholesterol involves rearrangements of the double bonds within the sterol rings. A detailed review describing cholesterol biosynthetic reactions is available (30).

Recent work by Mitsche et al. shows that utilization of the Bloch pathway or Kandutsch-Russell pathway is cell and tissue type-dependent (29). No tissues use canonical Kandutsch-Russell pathway, but instead may use a proposed “modified” Kandutsch-Russell pathway, in which DHCR24 mediates the entry of zymosterol into Kandutsch-Russell pathway (Figure 1). In mice, the Bloch pathway mediates 90% or more cholesterol biosynthesis in the testis, spleen, and adrenal, and the modified Kandutsch-Russell pathway is used for more than 70% of cholesterol biosynthesis in the brain, skin, and preputial (29).

### Regulation of sterol synthesis

Mammalian cells acquire cholesterol from two sources: endogenous synthesis and uptake from exogenous sources. These two processes are tightly controlled for cellular cholesterol content maintenance by multiple modes of regulation at transcriptional and post-transcriptional levels.

SREBP transcription factors serve as master regulators of cholesterol synthesis [reviewed in (31)]. There are two SREBP genes (*SREBF1* and *SREBF2*) and three SREBP proteins, SREBP-1a, SREBP-1c, and SREBP-2. *SREBF1* encodes SREBP-1a and SREBP-1c through alternative splicing, and *SREBF2* encodes SREBP-2 protein [reviewed in (32)]. SREBP-2 regulates expression of virtually all genes involved in cholesterol synthesis. SREBP-1a regulates both cholesterol and fatty acid synthesis, whereas SREBP-1c participates in the regulation of fatty acid synthesis [reviewed in (32)]. SREBPs are unique, membrane-bound transcription factors. When cellular cholesterol is at sufficient levels, SREBPs locate at the ER as inactive forms by forming a complex with SCAP and INSIG. INSIG1 and INSIG2 are ER resident proteins, and act as retention factors of the SCAP-SREBP complex in the ER. INSIG proteins are stabilized by side-chain oxysterols including 25-OHC and 27-OHC through direct interaction with the oxysterols (18). Upon cellular cholesterol reduction, the SCAP-SREBP complex is transported to the Golgi via the COP-II pathway, and SREBPs are sequentially cleaved by two proteases, Site-1 protease (S1P) and Site-2 protease (S2P), to liberate the transcriptionally active domain from membranes. SCAP possesses a sterol-sensing domain (SSD) and can bind cholesterol. Cholesterol binding to SCAP leads to a conformational change, which prevents the SCAP-SREBP complex from its incorporation into the COP-II vesicles (13, 33). Moreover, small changes in ER cholesterol levels regulate this translocation: SCAP-SREBP-2 complex leaves the ER for the Golgi when the ER cholesterol concentration is below 5% of total ER lipids (34). In addition, polyunsaturated fatty acids regulate proteolytic processing of SREBP-1, but not SREBP-2 (35).

In addition to transcriptional regulation, several important enzymes are post-transcriptionally regulated to control cholesterol synthesis. The best-characterized enzyme is HMGCR, an ER-bound protein with eight transmembrane domains and a catalytic domain that projects into the cytosol. As the rate-limiting cholesterol synthesis enzyme, HMGCR has multiple modes of regulation. In addition to SREBP-dependent transcriptional control, HMGCR is regulated post-transcriptionally. HMGCR is a short-lived protein with a half-life of 1 h when cellular cholesterol is at sufficient levels (36). HMGCR is degraded by the ubiquitin-proteasome pathway; ubiquitination of HMGCR requires INSIG-1 or-2 (37). INSIG proteins bind not only SCAP but also HMGCR via their SSD (38). INSIG binding to HMGCR facilitates the ubiquitination and degradation. HMGCR ubiquitination is promoted by the intermediate sterol dihydrolanosterol, and the side-chain oxysterols 25-OHC and 27-OHC, but not by cholesterol itself (10, 11, 39). Geranylgeraniol further accelerates degradation of HMGCR in the presence of sterols, but has little effect on the degradation by itself, showing a synergistical effect with sterols (39). Side-chain oxysterols stabilize INSIG proteins that promote HMGCR degradation. Three membrane-bound E3 ligases for HMGCR, gp78 (40), TRC8 (41), and RNF145 (42) have been identified. Importantly, all of these E3 ligases can interact with INSIG-1 and/or INSIG-2 (gp78 binds only INSIG-1), which stimulate HMGCR ubiquitination and degradation. However, involvement of gp78 and TRC8 in sterol-dependent HMGCR degradation may need further clarification since conflicting results have been reported (43). Additionally, another E3 ligase MARCH6 may also be involved in the regulation of HMGCR protein abundance (44).

In addition, phosphorylation regulates enzymatic activity of HMGCR. Serine-872 in human HMGCR (or serine-871 in mouse and hamster HMGCRs) is phosphorylated by AMP kinase (AMPK), a protein serine/threonine kinase regulated by cellular AMP levels (45). This phosphorylation inactivates the enzyme activity. Phosphorylation-dependent inactivation and sterol-dependent degradation are independently regulated to meet cellular demands (46).

In addition to HMGCR, recent studies identified squalene monooxygenase (47) and 7-dehydrocholesterol reductase (48) as enzymes highly regulated at post-transcriptional levels. Both enzymes are rapidly degraded by the ubiquitin-proteasome pathway in response to cholesterol loading (47, 48). The E3 ligase MARCH6 mediates the degradation of squalene monooxygenase (44).

### Esterification and hydroxylation of cholesterol

**Figure 2:**
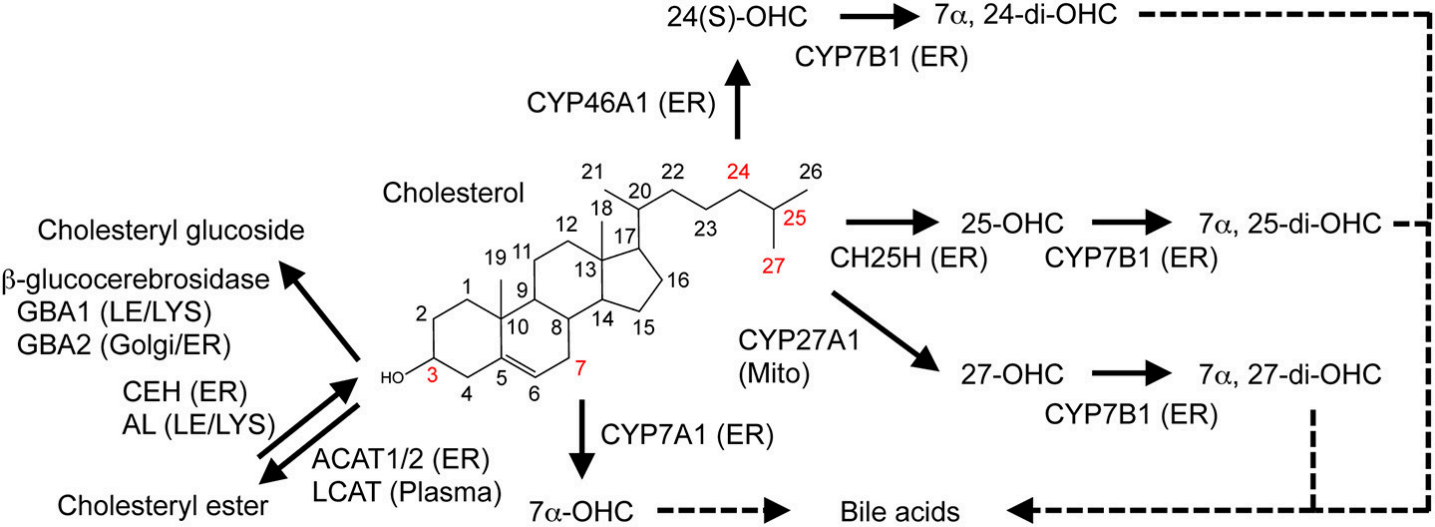
Metabolic conversion of cholesterol. Enzymatic conversion of cholesterol into oxysterols, cholesteryl ester, and cholesteryl glucoside is shown. Enzymes responsible for the conversion and their localization are also indicated. See text for more details. ER, endoplasmic reticulum; LE, late endosome; LYS, lysosome; Mito, mitochondria.

Because cholesterol cannot simply be degraded, excess cholesterol undergoes enzymatic esterification and hydroxylation within cells (Figure 2). To prevent toxic accumulation of free cholesterol, excess cholesterol is esterified, and stored in lipid droplets [reviewed in (49)]. This esterification is catalyzed by the ER-resident enzyme acyl-CoA:cholesterol acyltransferase 1 (ACAT1, also known as sterol O-acyltransferase 1, SOAT1), which transfers various long chain fatty acids such as oleic acid to the 3β-position of cholesterol (49). ACAT1 is ubiquitously expressed, while ACAT2, another form of ACAT, is mainly expressed in the intestine (enterocytes) and the liver (hepatocytes). In plasma, lecithin:cholesterol acyltransferase (LCAT) esterifies cholesterol in high-density lipoprotein (HDL). Cholesterol esterification is reversible; cholesteryl ester hydrolase located in the ER converts CE to cholesterol (8, 50), while acid lipase mediates hydrolysis of lysosomal CE derived from low-density lipoprotein (LDL). In addition to cholesterol, oxysterols (such as 24(*S*)-OHC, 25-OHC, and 27-OHC), pregnenolone, and phytosterols are substrates of ACAT1 [reviewed in (51)]. Intermediate sterols containing gem-dimethyl moieties at C4 position cannot act as ACAT substrates because the dimethyl moieties sterically hinder the 3β-OH group (52).

Enzymatic and non-enzymatic processes oxidize cholesterol [reviewed in (53)]. It is converted to oxysterols by the addition of one or more hydroxyl groups, keto groups, or epoxy groups. Side-chain oxysterols act as a potent negative regulator of cholesterol biosynthesis. Recent studies showed that cholesterol can also be glucosylated by β-glucocerebrosidase (GBA1 and GBA2), forming β-cholesteryl glucoside (Figure 2) in response to heat shock [reviewed in (54)]. In this review, we focus on the enzymatic production of the cholesterol metabolites, oxysterols, and discuss the roles of several major oxysterols and related enzymes. In mammals, many enzymes mediate cholesterol hydroxylation [reviewed in (53)]. With one exception, cholesterol 25-hydroxylase CH25H, cholesterol hydroxylases belong to the cytochrome P450 family. CH25H is a member of proteins that use diiron-oxygen as a cofactor (55). Hydroxylation of cholesterol occurs on its side-chain and/or on its steroid B ring. Here we review several major oxysterols that play important roles in human health and diseases.

25-OHC is biosynthesized from cholesterol via CH25H, a membrane-bound enzyme localized to the ER orienting the catalytic domain to the ER lumen. 25-OHC is also enzymatically synthesized by cholesterol 27-hydroxylase (CYP27A1) and cytochrome P450 3A4 (CYP3A4) and can be generated by free radical oxidation (53). 25-OHC can be metabolized by CYP7B1 (25-hydroxycholesterol 7α-hydroxylase), an enzyme that adds an OH-group to the steroid ring (at C7 position) in the ER, generating 7α, 25-dihydroxycholesterol (7α,25-di-OHC), a bile acid precursor (17) as well as a ligand for the G-protein-coupled receptor EBI2 (22, 23).

27-OHC is generated via cholesterol 27-hydroxylase (CYP27A1), which resides in the mitochondria. CYP27A1 can also convert cholesterol to 25-OHC. PM cholesterol is transported to the mitochondria where it serves as a major substrate for this hydroxylase (56). 27-OHC is the most abundant oxysterol in the plasma. CYP27A1 is expressed in many tissues including the liver, lung, and small intestine. Like 25-OHC, 27-OHC is further hydroxylated by CYP7B1 at the C7 position in the ER, forming 7α, 27-di-OHC that serves as a bile acid precursor (17).

Cholesterol 24-hydroxylase (CYP46A1) catalyzes biosynthesis of 24(*S*)-OHC [reviewed in (57)]. This enzyme is highly expressed in the brain, particularly in neurons. In the human brain, 24(*S*)-OHC is present at high concentration (up to 15 ng/mg wet weight) (58). Excess cholesterol in the brain is converted to 24(*S*)-OHC, which is then released into the plasma across the blood-brain barrier. Thus the conversion of cholesterol to 24(*S*)-OHC is an important step for eliminating excess cholesterol from the brain [reviewed in (57, 59)].

Hydroxylation of the cholesterol C7 position is catalyzed by CYP7A1 (cholesterol 7α-monooxygenase or cholesterol 7α-hydroxylase), which is almost exclusively expressed in the liver. CYP7A1 produces 7α-OHC, the major precursor of bile acids, and is a rate-limiting enzyme for synthesizing bile acids [reviewed in (17)].

### Regulation of oxysterol synthesis

Although much less is known about regulation of oxysterol synthesis, a growing body of evidence has shown that enzymes involved in hydroxylation of cholesterol is also subjected to tight regulation. Expression of CH25H is highly transcriptionally regulated. In macrophages and hepatocytes, virus infection leads to upregulation of CH25H mRNA levels and to marked increases in 25-OHC (60–63). Furthermore, lipopolysaccharide (component of gram-negative bacteria) induces its expression through toll-like receptor 4 in macrophages (64, 65). Signal transducer and activator of transcription factor 1 (STAT1), a transcription factor activated by type I interferon signaling, induces Ch25h mRNA expression (61). The resultant product 25-OHC has an anti-viral role by suppressing virus infection and replication.

CYP27A1 expression is not associated with cellular cholesterol levels in macrophages, but its expression is transcriptionally regulated by several nuclear hormone receptors. In human macrophages, retinoid X receptor (RXR) and peroxisome proliferator-activated receptor-γ (PPARγ) cooperatively induce expression of CYP27A1 mRNA (66). In HepG2 or Huh7 human hepatoblastoma cells, CYP27A1 expression is increased by glucocorticoids, growth hormone, and insulin-like growth factor 1, and decreased by cholic acid and thyroid hormones (67, 68). In human intestinal Caco2 cells (a colon adenocarcinoma cell line) but not in human hepatocytes, pregnane X receptor (PXR) induces CYP27A1 expression (69). Collectively, CYP27A1 expression is differentially regulated in a cell type-dependent manner.

Expression of CYP7A1, the rate-limiting enzyme of bile acid synthesis, is positively regulated by cholesterol and negatively by bile acids in the liver. Several nuclear receptors including LXRα, farnesoid X receptor (FXR), small heterodimer protein (SHP-1), and liver receptor homolog-1 (LRH-1) cooperatively control CYP7A1 transcription (70, 71). Upon an increase in intake of dietary cholesterol, LXRα activates CYP7A1 gene expression and facilitates bile acid synthesis for excretion in rodents (72). LXR does not activate CYP7A1 expression in humans due to lack of an LXR response element in the human *CYP7A1* promoter (73, 74). For transcriptional repression, FXR binds bile acids, inducing the expression of SHP-1, a nuclear receptor with promoter-specific repressor activity that suppresses CYP7A1 expression by inhibiting LRH-1, a nuclear receptor that positively regulates CYP7A1 expression in humans and rodents (70, 71).

## Intracellular sterol transport and cholesterol homeostasis

**Figure 3:**
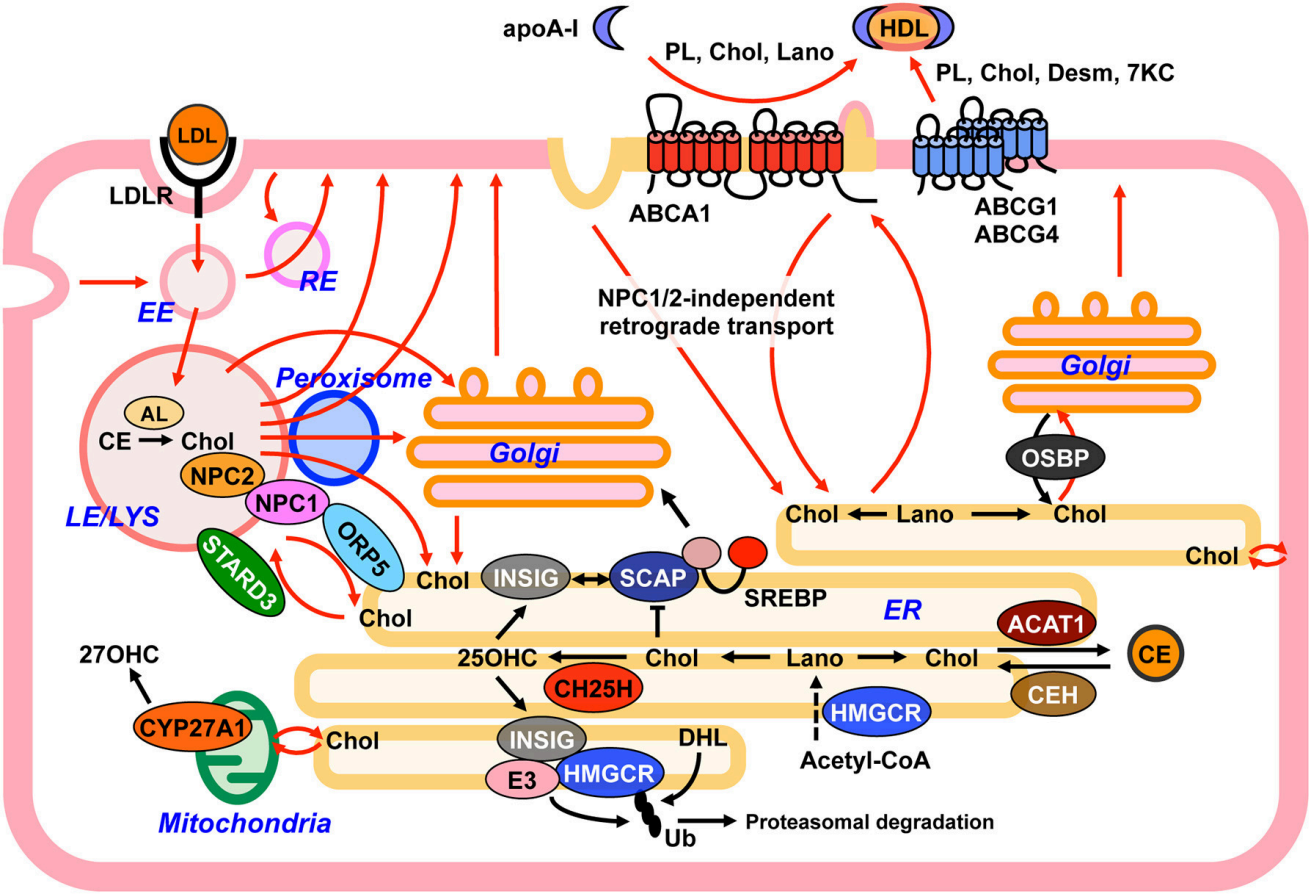
Model depicting intracellular sterol movement. Within cells, sterols dynamically move among organelles (red lines) to maintain cholesterol homeostasis. See text for more details. 7KC, 7-ketocholesterol; 25OHC, 25-hydroxycholeterol; 27OHC, 27-hydroxycholeterol; AL, acid lipase; CE, cholesteryl ester; Chol, cholesterol; Desm, desmosterol; DHL, dihydrolanosterol; EE, early endosome; ER, endoplasmic reticulum; Lano, lanosterol; LE, late endosome; LYS, lysosome; PL, phospholipid; RE, recycling endosome; Ub, ubiquitin.

Cholesterol moves dynamically within a cell (Figure 3), with this movement being essential to control cellular cholesterol homeostasis and to regulate heterogeneous cholesterol distribution among organelles (7, 8). The PM contains most of the cellular cholesterol. Cholesterol is also abundant in the endocytic compartments and the *trans*-Golgi network (TGN). In contrast, the ER and the mitochondria contain only 1% (or less) of total cellular cholesterol. How mammalian cells maintain uneven cholesterol distribution among organelles remains largely unknown. Differences in lipid compositions of each organelle may affect cholesterol-lipid interaction in membranes, allowing for organelle-specific cholesterol contents (4). As sterol is a highly hydrophobic substance, its transport between organelles, probably even within an organelle, requires transport carriers. Both vesicular (membrane-based) and non-vesicular (protein-based) transport are involved in intracellular transport of sterols.

### Sterol transport proteins with sterol binding ability

Several proteins directly interact with sterols and transport them within cells. In patients with Niemann-Pick type C (NPC) disease, a lysosomal storage disorder with fatal neurodegeneration, either NPC1 or NPC2 protein is defective (75). Defects in NPC1 or NPC2 cause the aberrant accumulation of unesterified cholesterol and sphingolipids in the late endosome (LE) and the lysosome (LYS) (75). NPC1 and NPC2 both locate to the LE/LYS, but their localization within the organelles is different: NPC1 has multiple transmembrane domains and localizes to LE/LYS membranes, while NPC2 is a soluble protein and localizes to the lumen. Both NPC1 and NPC2 can directly bind cholesterol, and cooperatively contribute to exporting cholesterol from the LE/LYS as described below.

Oxysterol-binding protein (OSBP) and OSBP-related proteins (ORPs) constitute a family of proteins that have the ability to bind sterol and other lipids [reviewed in (76, 77)]. In humans, 12 members, OSBP and ORP1-11, belong to this family, producing 16 different proteins through alternative splicing. In yeast, there are seven OSBP/ORP proteins, Osh1-7. The OSBP-related domain (ORD that consists of a hydrophobic pocket) is conserved in all OSBP/ORP proteins. Only two members, ORP5 and ORP8 contain transmembrane domain at their C-terminal region. Other functionally important domains found in most, but not all OSBP/ORPs include a pleckstrin homology (PH) domain and a FFAT (diphenyl alanine in an acidic tract) domain. OSBP was originally found as a protein that has high affinity to 25-OHC, in 1980s (78). Recent studies demonstrated that OSBP transports cholesterol (79, 80). A growing body of evidence [reviewed in (77)] has revealed that OSBP/ORPs transport not only sterols but also other lipids including phosphatidylserine and phosptidylinositol 4-phosphate at membrane contact sites (MCSs), which are proximal regions of two organelle membranes, through non-vesicular process.

Other sterol transport proteins belong to steroidogenic acute regulatory (StAR) protein-related lipid transfer (START) domain family, which consists of 15 members, STARD1-15 [reviewed in (81)]. The START domain forms a hydrophobic cavity that can accommodate one lipid molecule. STARD proteins also mediate non-vesicular lipid transport, and some of these proteins can bind sterols.

### Anterograde transport of cholesterol and intermediate sterols

The enzymes responsible for converting squalene to cholesterol are all located at the ER, indicating that intermediate sterols and cholesterol are synthesized in this organelle. Upon synthesis, cholesterol leaves the ER, and rapidly reaches the PM with a half-time of 10–20 min (82, 83). In addition to cholesterol, cells contain small but significant amounts of intermediate sterols as cholesterol precursors. A significant portion of intermediate sterols including lanosterol (C30 sterol), dimethylsterols (C29 sterols), monomethysterols (C28 sterols), zymosterol (C27 sterol), and desmosterol (C27 sterol) is transported to the PM immediately after synthesis but prior to conversion to cholesterol (84–87). The ER-to-PM anterograde sterol transport is not impaired in NPC cells (87), indicating that an NPC1/NPC2-independent pathway transports sterols from the ER to the PM. Involvement of OSBP in the anterograde sterol transport has been recently suggested (79). Upon binding to 25-OHC, OSBP translocates to the Golgi apparatus from cytoplasm (88). Recent findings show that OSBP counter-exchanges cholesterol in the ER and phosphatidylinositol 4-phosphate (PI4P) in the Golgi (80). On the other hand, an earlier study showed that there are Golgi-dependent and Golgi-independent routes that transport newly synthesized cholesterol from the ER to the PM, and that the Golgi-independent route plays a major role in this anterograde transport (83). How much newly synthesized cholesterol relies on OSBP for reaching the PM is unknown. Additionally unknown and of interest is whether OSBP can transfer intermediate sterols from the ER to the Golgi. In addition to OSBP, several other proteins including ORP2 (89) and sterol carrier protein-2 (90) are suggested to participate in the anterograde transport; however, this involvement needs further investigation.

### Active cholesterol hypothesis, sterol sensing, and sterol homeostasis

At the PM, cholesterol forms stoichiometric complexes with phospholipids, particularly with SM and glycerophospholipids containing long saturated acyl chains (91). Cholesterol pools that exceed the binding capacity of phospholipids could become more chemically active and more mobile. This mobile cholesterol is recognized as “active” cholesterol, and is expected to act as regulatory cholesterol for cellular cholesterol homeostasis (92). The ER contains a series of sterol sensing proteins. For storage, excess cellular cholesterol is esterified by the ER resident enzyme ACAT1 (49). ACAT1 enzymatic activity is stimulated by cholesterol and oxysterols. As described above, SREBP activation depends on the transport of SCAP-SREBP complex form the ER to the Golgi via COPII pathway. This transport is sensitive to ER cholesterol levels (34). A novel ER sterol sensing protein was recently identified; nuclear factor erythroid 2 related factor-1 (Nrf1, also known as Nfe2L1) is an ER-membrane bound transcription factor, which is cleaved near its N-terminus to be released from the ER. Through its ability to bind cholesterol, cholesterol blocks the cleavage and translocation of Nrf1 to the nucleus, and enhances LXR target gene expression including *Abca1* and *Abcg1* as a means to respond to excess cellular cholesterol (93). Thus, delivering active cholesterol to the ER is considered an essential step to sense and respond to excess cellular cholesterol (94, 95). Retrograde sterol transport from the PM to the ER involves both vesicular and non-vesicular transport. Here we describe the current understanding of these two transport processes and the proteins involved.

### Retrograde sterol transport

Cells can acquire exogenous cholesterol from lipoproteins. The best-characterized, major lipoprotein uptake process is LDL receptor (LDLR)-mediated internalization of LDL (Figure 3). LDL contains a large amount of cholesterol in an esterified form. LDL is first bound at the cell surface by LDLR, and internalized by endocytosis (96). LDLR-mediated endocytosis is classified as clathrin-mediated endocytosis (CME). CE in LDL is hydrolyzed by acid lipase (Figure 2) in endocytic compartments, yielding free cholesterol (97). Endogenously synthesized cholesterol and a portion of intermediate sterols are also internalized from the PM and reach the LE/LYS (87, 98). Egress of free cholesterol from the LE/LYS requires the two cholesterol-binding proteins, NPC1 and NPC2. NPC1 is a large membrane-bound protein with 10 transmembrane domains (TMDs), whereas NPC2 is relatively small, 154-amino acid protein localized to luminal side of the LE/LYS. NPC1 has a cholesterol-binding pocket in its N-terminal luminal side (99). NPC1 also contains a SSD. Although whether the SSD is also able to directly bind cholesterol is not known, mutations within this domain lose cholesterol-transporting and cholesterol-binding activities (100). A potential model by which NPC1 and NPC2 act in concert to export cholesterol from the LE/LYS has been proposed (99, 101). In this “hand-off” model, at luminal side of the LE/LYS, NPC2 binds cholesterol and transfers it to the N-terminal, luminal cholesterol-binding domain of NPC1. Cholesterol is then incorporated into late endosomal/lysosomal membranes, and moves to various organelles including the ER, TGN (102), PM, mitochondria (103), and peroxisome (104) in a manner dependent of NPC1 and NPC2. In response to LDL uptake, delays in SREBP inactivation and in cholesterol re-esterification by ACAT1 both at the ER are observed in NPC cells. In addition, deficiency in either NPC1 or NPC2 impairs the conversion of LDL-derived cholesterol to 25-OHC at the ER and 27-OHC at the mitochondria (103). LDL-derived cholesterol also reaches the PM in a manner dependent on NPC1/NPC2 activity and becomes available for ABCA1-dependent release (105). As such, NPC1/NPC2 proteins play a crucial role in redistribution of internalized cholesterol from the LE/LYS to other organelles.

Both vesicular and non-vesicular transport processes participate in cholesterol transport from the LE/LYS to other organelles. Vesicular cholesterol transport to the ER involves several TGN-specific SNARE proteins including syntaxin 6, syntaxin 16, and VAMP4, indicating that LDL-derived cholesterol is at least partially delivered to the TGN from the LE/LYS before arriving at the ER (102). Transport of LDL-derived cholesterol from the LE/LYS to the PM is mediated by Rab8-dependent vesicular trafficking (106). Non-vesicular cholesterol transport is at least partly mediated by MCS. At the LE/LYS-ER MCS, ORP5 binds to NPC1, and transport cholesterol from the LE/LYS to the ER (107). On the other hand, at the same MCS, STARD3 mediates cholesterol transport from the ER to the LE/LYS (108). Chu et al. found that significant amounts of cholesterol in the LE/LYS are transported to peroxisomes through LE/LYS-peroxisome MCS, which precedes its arrival at the ER and PM (104). The LE/LYS physically interacts with peroxisomes, and the MCS formation is mediated by synaptotagmin VII at the LE/LYS and PI(4,5)P2 at peroxisomes (104). Unknown cytosolic factors are suggested to facilitate cholesterol movement from the LE/LYS to peroxisomes (104).

Retrograde sterol transport from the PM to the ER is an important element of sterol sensing at the ER (95). Aside from NPC1/NPC2-dependent pathway, recent works show that mammalian cells utilize a PM-to-ER sterol trafficking route that does not require NPC proteins (87, 109). The nature of this NPC1/NPC2-independent sterol transport is not well understood, but it has been suggested that both vesicular and non-vesicular mechanisms participate in it. ER membranes are partly elongated to proximal regions of the PM and form the MCSs where non-vesicular lipid transport can occur. The ORP proteins ORP1S and ORP2 are implicated in the PM-to-ER cholesterol transport through a non-vesicular pathway (110). Other studies show that STARD4 mediates cholesterol transport from the PM to the endocytic recycling compartments and to the ER (111). In addition to a non-vesicular process, involvement of endocytic sterol internalization is also proposed as a mechanism for the delivery of PM sterol to the ER (87, 94). Inhibition of clathrin-independent endocytosis (CIE), but not CME, significantly impairs PM-to-ER transport of cholesterol and intermediate sterols (87, 94). This CIE is suggested to require dynamin, a GTPase that plays a key role in scission of endocytic structure from the PM. Importantly, blocking dynamin activity results in a marked increase in SREBP-2 processing and in a reduction in esterification of PM cholesterol, indicating a decrease in ER cholesterol levels (94).

ABCA1, which plays an essential role in HDL formation (discussed in more detail in the following section), mediates transport of phospholipids and sterols. Although ABCA1 transports lipids to apoA-I for formation of HDL, its activity is independent of availability of lipid acceptors such as apoA-I (112, 113). In absence of a lipid acceptor, ABCA1 deficiency represses retrograde cholesterol transport (94), causes accumulation of cholesterol in the PM (94, 114), and impairs internalization of cholera toxin B subunit, a marker of CIE (94). It is therefore plausible that ABCA1 contributes to the regulation of PM lipid composition and functionality of PM through its lipid transport activity. How and whether lipid compositions of the PM are modulated by ABCA1 and other lipid transporters, and whether this affects PM functionality as well as non-vesicular sterol transport are important issues yet to be determined.

Select tissues including the liver and steroidogenic tissues also acquire cholesterol from plasma HDL for biliary secretion and steroidogenesis, respectively (115). Scavenger receptor class B type I (SR-BI) serves as a HDL receptor, and mediates the selective uptake of HDL-cholesterol (both CE and free cholesterol) (116). In contrast to the endocytic internalization of LDL, SR-BI-mediated uptake does not involve lysosomal degradation of HDL. How HDL-cholesterol is internalized and transported into cell interior through SR-BI remain to be clarified (117).

### Intracellular transport of oxysterols

Oxysterols are synthesized in the ER and mitochondria depending on hydroxylase localization. Oxysterols play various roles in different organelles. Oxysterols bind LXR, and regulate the transcription of LXR target genes in the nucleus. At the ER, side-chain oxysterols bind INSIGs, leading to their stabilization (18), and also become ACAT1 substrates (51). At the PM, oxysterols can affect membrane organization (27). Oxysterols are also released from cells (118). Furthermore, several proteins with sterol transporting activity, including OSBP (78) and NPC1 (119) have the ability to bind oxysterols. Together these findings strongly suggest that, like cholesterol, oxysterol distribution is tightly regulated. However, little is known about intracellular transport of oxysterols due to their extremely low cellular contents (~0.1% of cholesterol), in addition to difficulties in their handling.

Recent work developed an intrinsically fluorescent oxysterol, 25-hydroxycholestatrienol (25-HCTL) that mimics 25-OHC (120). 25-HCTL is a hydroxylated derivative of cholestatrienol (CTL), a fluorescent cholesterol analog without fluorophore that may affect the physical properties of cholesterol. Both 25-HCTL and CTL contain two additional double bonds at the steroid B and C rings, thereby exhibiting intrinsic fluorescence with excitation max at 325 nm. When cells are incubated with 25-HCTL in the presence of LDL, 25-HCTL enters cells via LDLR-mediated endocytosis, and is transported to the LE/LYS. Like cholesterol, egress of 25-HCTL from the intracellular compartments requires functional NPC1 protein (120), which is consistent with 25-OHC-binding ability of NPC1 protein (119). Furthermore, 25-HCTL can be transported to the ER, and is also redistributed to the PM and the recycling endosome (120). It remains to be determined how endogenously synthesized oxysterols are transported from the sites where they are synthesized: the ER and mitochondria.

## Sterol export to extracellular milieu

Because mammalian cells cannot break down the sterol backbone, and conversion of cholesterol into bile acids is restricted to only the liver, efflux of excess cholesterol and other sterols from cells is crucial for cellular cholesterol homeostasis. Cholesterol can be removed from cells by two processes; active export and passive diffusion. Passive diffusion largely depends on a cholesterol gradient between the cell surface and cholesterol acceptors such as HDL. Active export is energy-dependent and involves several ABC transporter proteins that use ATP as driving force.

### ABC transporters

The human genome encodes 48 ABC transporters, classified into seven subfamilies, ABCA-G families (121). ABC transporters bind and hydrolyze ATP for energy-dependent transport of chemically diverse substrates across biological membranes. The substrates vary greatly for the various ABC transporters. Studies have revealed that several ABC transporters play a role in exporting cellular sterols including cholesterol, intermediate sterols, oxysterols, and phytosterols (122), as described in further detail in this review.

### ABCA1 and HDL assembly

It was first reported in 1991 by Hara and Yokoyama (123) that interaction of lipid-free apoA-I with macrophages resulted in formation of HDL with cellular phospholipids and cholesterol. It was subsequently demonstrated that fibroblasts isolated from patients with Tangier disease, a severe HDL deficiency, lack the apoA-I-mediated phospholipid and cholesterol release and HDL formation (124). In 1999, *ABCA1* was identified as the gene mutated in Tangier disease patients (125–127). These reports and numerous other studies established that ABCA1 plays an essential role in the release of cellular phospholipids and cholesterol and in the assembly of nascent HDL [reviewed in (128)]. Further studies with conditional *Abca1* knockout mice revealed that liver ABCA1 and intestinal ABCA1 contribute to produce 70–80 and 30% of total plasma HDL, respectively (129, 130). The physiological acceptor for phospholipids and cholesterol is apoA-I, a major HDL apolipoprotein. HDL is a key lipoprotein that transports excess cholesterol from peripheral tissues to the liver where cholesterol is converted to bile acids for excretion. This cholesterol transport system is often referred to as “reverse cholesterol transport.” Because Tangier disease patients largely lack serum HDL, they exhibit accumulation of cholesterol (both unesterfied and esterified cholesterol) in peripheral tissues and have increased risk of cardiovascular disease (131–133).

How does ABCA1 mediate HDL formation? ABCA1 localizes to both the PM and the endocytic compartments. ABCA1 that resides in the PM mediates the assembly of nascent HDL (134), while ABCA1 internalized into the endocytic compartments is subjected to degradation or recycles back to the PM (135). ABCA1 creates membrane deformation sites at the PM, where apoA-I binds (136). This could be related with the finding that overexpression of ABCA1 disrupts lipid rafts (137). In contrast, ABCA1 deficiency increases lipid rafts and cholesterol contents in the PM (94, 114, 138). ApoA-I can directly bind to ABCA1, and this interaction plays a role in ABCA1-dependent lipid release and HDL formation; therefore, lipid compositions of nascent HDL may reflect those of membrane domains where ABCA1 mediates HDL assembly. A study using recombinant ABCA1 reconstituted in liposomes shows that ABCA1 can export phosphatidylcholine (PC), phosphatidylserine (PS), and SM (139). Cholesterol reduces ATPase activity of ABCA1 (112, 139), suggesting that cholesterol may not be a direct substrate of this transporter. These *in vitro* observations are consistent with a cell-based study showing that ABCA1 primarily mediates phospholipid release to apoA-I. Recent work showed that ABCA1 also flops and releases phosphatidylinositol (4,5) bis-phosphate (PIP2) (140). Increases in cell surface PIP2 levels enhances apoA-I binding to cell surface and ABCA1-dependent HDL biogenesis (140). In certain cell models, ABCA1 primarily releases phospholipids and can generate cholesterol-poor nascent HDL particles (141). ABCA1-dependent cholesterol release can thus uncouple phospholipid release and HDL formation. Although the mechanisms by which ABCA1 mediates cholesterol incorporation into nascent HDL remain obscure, cholesterol availability for ABCA1-dependent HDL formation is dependent on cellular cholesterol pool sizes regulated by ACAT1 (142). In cholesterol-loaded macrophages, autophagy-dependent lysosomal degradation of lipid droplets, called lipophagy, hydrolyzes lipid droplet-associated CE by lysosomal acid lipase, generating free cholesterol available for ABCA1-dependent HDL assembly (143).

Our recent work determined sterol specificity of ABCA1-mediated sterol release (144). We showed that in addition to cholesterol, ABCA1 preferentially releases intermediate sterols with extra methyl groups including lanosterol, which accounts for very minor quantity of cellular sterol (0.1% or less of cellular total cholesterol) and its half-life is <1 h in cells. Lanosterol and other methylated intermediate sterols synthesized at the ER are immediately delivered to the PM (87). Therefore it is postulated that these minor sterols become constituents of certain PM domains where ABCA1 preferentially release these sterols along with cholesterol and phospholipid for HDL formation (144). There are significant structural differences between lanosterol and cholesterol, and lanosterol has the ability to induce membrane curvature formation in model membranes (145). Whether lanosterol affects membrane structure/organization in living cells remains to be investigated. In the absence of acceptors, lanosterol and other intermediate sterols immediately leave the PM domains and are transported back to the ER for cholesterol synthesis (87). ABCA1 participates in this retrograde transport (94). ABCA1 deficiency thus associates with accumulation of cholesterol and other sterols in the PM of various cell types including macrophages and fibroblasts (94, 114). In addition to endogenously synthesized sterols, LE/LYS-derived cholesterol is a significant source for ABCA1-dependent HDL formation (105, 146).

Recently, the structure of human ABCA1 in digitonin micelles but not in phospholipid bilayer was determined by cryoelectron microscopy (cryo-EM) (147). The structure reveals that an elongated hydrophobic tunnel is present within the two large extracellular domains. Whether this hydrophobic tunnel serves as a route for delivering lipids to apoA-I needs further investigation. Although the molecular mechanisms by which ABCA1 transports lipid and mediates HDL biogenesis remain obscure, the cryo-EM structure supports that structure-based studies of ABCA1 can provide further insights into its function and HDL assembly.

### Regulation of ABCA1

ABCA1 expression is tightly regulated at transcriptional and post-transcriptional levels. LXR serves as major transcription factor that activate *ABCA1* gene expression (148, 149). Oxysterols and intermediate sterols, including desmosterol, activate LXR and induce ABCA1 expression (14, 148). LXR forms a heterodimer with another nuclear receptor, RXR. 9-*cis* retinoic acid, an RXR ligand, also induces ABCA1 expression (148). Recent studies found that many microRNAs (miRNAs), such as miR-33a/b and miR-148 are involved in *ABCA1* gene expression [reviewed in (150)]. These miRNAs suppress *ABCA1* gene expression and reduce plasma HDL levels in mice and non-human primates (151, 152, 153). MiR-33a and miR-33b are encoded by introns of *SREBF2* and *SREBF1* genes, respectively (151, 152).

ABCA1 protein is a short-live protein with half-life of about 1–2 h (154, 155). ABCA1 is degraded by the calcium-dependent cysteine protease, calpain (154, 156), and in proteasomes or in the LYS through ABCA1 ubiquitination by an unknown ubiquitin ligase (157–159). Unsaturated fatty acids accelerate ABCA1 degradation (160). Free cholesterol loading enhances the ubiquination and degradation of ABCA1 (158, 161). Importantly, in addition to its lipid acceptor function, apoA-I has an ability to protect ABCA1 from calpain-mediated degradation, thereby increasing cell surface ABCA1 and further inducing lipid release (154, 156). It has been suggested that the ABCA1-apoA-I interaction at the cell surface leads ABCA1 to recycle back to the PM from the endocytic compartments (135) through a Rab8-dependent trafficking pathway (162). It would be interesting to determine whether the interaction between ABCA1 and apoA-I causes modifications of ABCA1 before endocytic internalization, to prevent its degradation.

ABCA1 has several important motifs/domains that modulate its stability. It has been demonstrated that the PDZ domain located in the C-terminal cytosolic region binds to several proteins including α1-syntrophin and β1-syntrophin (155, 163). Interaction of these two proteins with ABCA1 leads to its stabilization. LXRβ also binds to the C-terminal region of ABCA1 and stabilizes ABCA1 (164). The cytosolic region between the two transmembrane domains contains the PEST sequence that plays an important role in calpain-mediated degradation (154). Near the PEST sequence, ABCA1 contains calmodulin binding site. Calmodulin binding to ABCA1 inhibits ABCA1 degradation (165).

Several protein kinases including protein kinase A (PKA), PKC, and Janus kinase 2 (JAK2) modulate ABCA1 activity and stability. PKA phosphorylates ABCA1 and modulates its lipid efflux activity (166, 167). PKC, activated by apoA-I-cell interaction or by apoA-I-dependent lipid removal, is involved in the stability of ABCA1 (168, 169). JAK2 is activated by apoA-I and modulates ABCA1 activity without affecting its stability (170, 171). Other factors regulating ABCA1 protein activity and/or stability are reviewed elsewhere (172).

### ABCG1 and ABCG4

In addition to ABCA1, the ABCG subfamily proteins ABCG1 and ABCG4 are well-recognized members of ABC transporters that facilitate cholesterol efflux to HDL (173). ABCG transporters are half-transporters that contain one nucleotide-binding domain in the N-terminal region. ABCG1 and ABCG4 transporters form either homodimer or heterodimer to function as an active transporter. ABCG1 is expressed in numerous tissues. ABCG1 localizes to both the PM and the endocytic compartments when overexpressed (174, 175). At physiological expression levels, however, this transporter largely resides in the intracellular compartments and acts as intracellular cholesterol transporter (176). Regardless of its localization, deficiency of ABCG1 impairs cholesterol efflux to HDL in macrophages (177). Importantly, ABCG1 also exports 7-ketocholesterol, an oxysterol abundant in oxidized LDL and atherosclerotic lessions, to HDL, whereas ABCA1 does not (118). Because 7-KC can induce apoptosis in macrophages, ABCG1-mediated 7-KC efflux to HDL protects macrophages from its cytotoxicity (118). Furthermore, in addition to sterols, ABCG1 preferentially exports SM over PC (174). While how ABCG1 mediates efflux of sterols and phospholipids is poorly characterized, its ATPase activity is stimulated by cholesterol and SM *in vitro* (178). These findings suggest that the activities of ABCA1 and ABCG1 are differentially regulated. ABCA1 and ABCG1 cooperatively promote cholesterol export pathway, and as such act synergistically to prevent cholesterol accumulation in macrophages (179).

ABCG4 is highly expressed in the brain (180). ABCG1 and ABCG4 are expressed in both neurons and astrocytes. Both of these transporters have the ability to export cholesterol as well as the intermediate sterol desmosterol to HDL (180). Deficiency in ABCG1 and/or ABCG4 results in the accumulation of intermediate sterols including lanosterol, desmosterol, and lathosterol, but not cholesterol, in mouse brains, with greater accumulation in double knockout mice (180). In ABCG1/ABCG4 double knockout mice, 27-OHC but not 24(*S*)-OHC is also accumulated in the brain (180).

### ABCG5 and ABCG8

ABCG5 and ABCG8 form a heterodimer as an active transporter and are expressed in the apical membranes of enterocytes and hepatocytes. In the small intestine, ABCG5/G8 promote efflux of cholesterol and plant sterols absorbed through NPC1 like 1 (NPC1L1), to the lumen, thus limiting the absorption of dietary sterols (181). In the liver, the heterodimer exports cholesterol and phytosterols into the bile, thus enhancing sterol excretion from the body (182). Deficiency in either one the two transporters causes β-sitosterolemia, which is characterized by the robust accumulation of dietary sterols in plasma and tissues (183, 184). This accumulation is caused by increased intestinal absorption and decreased biliary excretion, and individuals with β-sitosterolemia develop premature atherosclerosis [reviewed in (185)].

### Other ABC transporters with potential sterol exporting activity

ABCA7, a close homolog of ABCA1, is highly expressed in the brain, lung, spleen, and adrenal in mice. ABCA7 is associated with Alzheimer's disease [reviewed in (186)]. The exact functions of ABCA7 are largely unknown, but it is capable of exporting lipids (phospholipid and cholesterol) to extracellular apoA-I and generates HDL-like particles when overexpressed in mammalian cells (187, 188). The ability of ABCA7 to export cholesterol is much less compared to that of ABCA1 (189). Consistent with the cell-based studies, an *in vitro* reconstitution assay shows that ABCA7 exports PS and PC with preference for PS (139). Although ABCA7 has similar lipid transporting functions as ABCA1, it plays a minimum, if any, role in lipid efflux and HDL formation in macrophages and in mice (190). At physiological expression levels, ABCA7 is mainly intracellular localized in resting macrophages, and following stimulation with apoptoic cells, ABCA7 translocates to the PM and plays a role in phagocytosis (191).

Additional ABCA family proteins are involved in cholesterol efflux. ABCA8 facilitates cholesterol efflux to apoA-I and modulates HDL-cholesterol levels (192). In humans, mutations in this gene are associated with low HDL-cholesterol levels (192). In addition, ABCA12 also regulates cholesterol efflux (193). It has been reported that ABCA12 interacts with ABCA1 and regulates its stability in macrophages, thereby modulating ABCA1-dependent lipid release (193). ABCA12 was originally identified as a protein defective in one of the most severe skin disorders, Harlequin ichthyosis that is characterized by severe skin barrier defects and by abnormal keratinocyte lamellar granules (194). It is suggested that ABCA12 is involved in the transport of glucosylceramide in the lamellar granules (194). Unlike other ABC transporters described above, ABCA2 attenuates cholesterol efflux to extracellular cholesterol acceptors through poorly characterized pathway (195).

Certain ABCB family proteins also exhibit cholesterol transport activities. ABCB1 (also known as multidrug resistant protein 1, MDR1, or P-glycoprotein) translocates cholesterol from the inner leaflet to the outer leaflet of the PM (196). ABCB4 (also known as MDR3 in humans or MDR3 in mice), is expressed on the apical canalicular membranes of hepatocytes and exports PC and cholesterol to bile acids, as such ABCB4 along with ABCG5/ABCG8 contributes to biliary cholesterol excretion (197).

### Passive diffusion

Cholesterol can be removed from cells through passive diffusion, which is driven by a cholesterol gradient between cell surface and acceptors [reviewed in (198, 199)], with HDL serving as the major cholesterol acceptor. LCAT has phospholipase A2 and acyltransferase activities; LCAT transfers an acyl-chain from PC to 3-OH position of cholesterol, resulting in the formation of CE. LCAT-mediated cholesterol esterification plays a role in HDL maturation (200). Cholesterol esterification by LCAT in HDL causes the expansion of the cores in HDL particles and the reduction of free cholesterol at HDL surface, further facilitating net cholesterol efflux from cells to HDL. Serum albumin acts as a shuttle to facilitate passive cholesterol efflux from cells (199). Although the HDL receptor SR-BI is known to enhance cholesterol efflux from cells to HDL, SR-BI-mediated cholesterol efflux may not contribute to net cholesterol removal from cells, as it mediates bidirectional transport of cholesterol between the cell surface and HDL [reviewed in (115)]. As previously described, SR-BI rather plays an important role in selective uptake of HDL-cholesterol in the liver and steroidogenic tissues (115).

## Sterols and human diseases

Cholesterol, sterol intermediates, and oxysterols play diverse and important roles in the body, in addition to contributing to various human diseases including cancer, cardiovascular disease, neurodegenerative disorders such as Alzheimer's disease and infection. This review focuses on discussing the connection between sterol metabolism and atherosclerosis as well as cancers. For a detailed discussion between cholesterol metabolism and neurodegenerative diseases, a recent review by Chang et al. (201) is available. Detailed reviews on the roles of sterols in immune responses are available elsewhere (20, 202). Pathophysiological roles of SREBPs in various human diseases are extensively discussed by Shimano and Sato (203).

### Sterols and atherosclerosis

Cholesterol along with inflammation regulation are major therapeutic targets for atherosclerosis, a condition in which plaque buildup in the artery wall can lead to a multitude of vascular complications, including heart attack and stroke. Large-scale trials have demonstrated the effectiveness of HMGCR inhibitors, statins, but have also shown residual risk with many statin-taking patients suffering from cardiovascular events (204). Further LDL cholesterol lowering by combining statin with the Niemann-Pick C1-like 1 intestinal cholesterol absorption inhibitor, ezetimibe provides modest benefit (205). Monoclonal antibody inhibition of proprotein convertase subtilisin-kexin type 9 (PCSK9), which reduces LDL cholesterol by about 60% via reducing LDL receptor degradation, improves clinical outcomes in patients with cardiovascular disease (206). While outcomes are not known, inhibition of the lipoprotein lipase inhibitor, angiopoitein-like 3 reduces circulating cholesterol, notably in homozygous familial hypercholesterolemia patients that only have limited responses to statin and PCSK9 therapies, and who typically require arduous LDL apheresis treatment (207). Supporting a key role of SR-BI-mediated HDL-cholesterol uptake in human reverse cholesterol transport, a loss-of-function variant (P376L) in SR-BI was recently identified in people with extremely high HDL cholesterol that also have increased risk of coronary heart disease (208). Much is known about the impact of reducing cholesterol in cardiovascular disease; however, less is known about the contributions of sterol metabolites to atherosclerosis that may provide insight into novel risk factors and therapies to target residual cardiovascular risk.

Macrophage lipid accumulation leads to foam cell formation and inflammation, contributing to atherosclerosis (209). In macrophages, the cholesterol precursor desmosterol exhibits several beneficial properties including inhibiting expression of cholesterol biosynthesis and proinflammatory genes, along with induction of LXR genes and cholesterol efflux (210). LXR-deficiency in mice exacerbates atherosclerosis disease pathology, and LXR activation generally reduces cell and animal model pathology through a variety of mechanisms [reviewed in (211)]; however, LXR activation can also have an accompanying unacceptable side effect of raising liver triglyceride synthesis (212).

Oxysterols are associated with nearly every atherosclerosis-contributing pathway, as such they present a promising area of future therapeutic research. Oxidized forms of cholesterol produced by LDL oxidation exhibit pro-atherosclerotic properties [reviewed in (213, 214)]. Like cholesterol, oxysterols can be stored as fatty acid esters [reviewed in (51)]. In mice, ACAT1 small molecule inhibition (215), genetic knockout (216), and macrophage-specific deletion (217) reduce atherosclerosis pathology. Additionally, ACAT1 was recently associated as a causal modifier variant explaining strain differences in mouse atherosclerosis pathology (218); although a cardiovascular benefit of ACAT inhibition has not been demonstrated in human ACAT inhibitor trials (219).

Oxysterols can regulate atherosclerosis-related pathologies, but the extent to which oxysterols drive atherosclerosis is unclear. Multi-omics mapping of oxysterols and associated genes and proteins in healthy and diseased tissue could clarify the complex roles of these lipids in cardiovascular disease. In primary mouse macrophages, 22-OHC, 24-OHC, and the sterol 24, 25 epoxycholesterol repress activation of the inflammation-related factor, inducible nitric oxide synthase, while also inducing ABCA1 expression, and 25-OHC and 27-OHC activate ABCA1 but do not repress inducible nitric oxide synthase (220). On top of plaque formation, calcification is frequently observed in atherosclerosis, and may contribute to plaque rupture (221). In bovine aortic and mouse smooth muscle cells, 25-OHC stimulates mineralization pathways leading to vascular calcification, including through LXR-independent mechanisms (222, 223). 27-OHC is increased in human atherosclerotic arteries and functions in macrophage cholesterol elimination (224); however, the role of 27-OHC in atherosclerosis is complex. Elevated 27-OHC via deletion of CYP7B1 that metabolizes 27-OHC promotes atherosclerosis via a proinflammatory process involving estrogen receptor alpha in apoE-deficient mice (225). Knockout of *Cyp27A1* in apolipoprotein E-deficient mice revealed a gene dose effect with a 10-fold reduction in atherosclerosis severity observed in double knockout mice, and *Cyp27A1* heterozygosity leads to accelerated atherosclerosis (226). Cerebrotendinous xanthomatosis develops in the absence of CYP27A1 in humans, while mice lacking CYP27A1 do not develop xanthomas. Further complicating matters are that loss of 27-hydroxylase may have athero-protective effects including increased cholesterol degradation, in addition to pro-atherogenic effects, such as reduced cholesterol efflux, and the balance of this may vary between patients (227). Raising a confounding factor, sex-specific effects in bile acids are observed in CYP27A1 overexpressing mice (227).

### Sterol metabolism and cancer

Altered metabolism is a hallmark of cancer cells, with a major metabolic alteration being elevated glucose uptake and glycolysis (228). In addition to this “glycolytic phenotype,” biosynthesis of lipids (including cholesterol), which are essential building blocks of cell membranes, is increased in cancer cells to support proliferation (229). Elevated lipogenesis is referred to as “lipogenic phenotype.” In human melanomas, increased expression of various cholesterol biosynthetic genes is found in more than 60% of patients (230). SREBPs serve as master drivers for lipogenic phenotype of cancer cells. Activation of SREBPs and/or upregulation of SREBP-target gene expression is observed in a wide variety of human cancers including glioblastoma (231), prostate cancer (232), breast cancer (233), melanoma (230, 234), and is often associated with poor prognosis/survival. Cholesterol is an indispensable component of lipid rafts, which serve as platforms for various oncogenic signals including Akt activation, and therefore cholesterol synthesis in cancer cells links to the integrity of these membrane domains. SREBPs are activated by PI3K-Akt-mTORC1 signaling (235, 236), which is hyperactivated in human cancers. Consequently, a positive feedback loop between PI3K-Akt-mTORC1 signaling and SREBP-dependent lipogenesis is formed to sustain malignant properties of cancer cells (234, 237). Several mechanisms are involved in the activation of SREBP by PI3K-Akt-mTORC1 signaling: (1) Phosphorylation of SREBP-1 mature form by the protein kinase GSK3β results in ubiquitination by the ubiquitin ligase Fbw7, leading to the proteasomal degradation of the mature form (238). Independently of mTORC1, Akt phosphorylates and inactivates GSK3β, thereby increasing SREBP transcriptional activity. (2) PI3K-Akt-mTORC1 axis regulates processing of both SREBP-1 and SREBP-2 (234). mTOR phosphorylates cytosolic CREB regulated transcription coactivator 2 (CRTC2) and attenuates its inhibitory effect on COPII-dependent transport of SREBP-1 from the ER to the Golgi, which facilitates SREBP-1 processing (239), and this pathway is at least partly involved in PI3K-Akt-mTORC1 regulation of SREBP processing. Whether this axis also regulates SREBP-2 processing is unknown at present. In addition, the mTOR-CRTC2 axis could be involved in general COP-II transport. Additional mechanisms may also participate in SREBP processing as the protein kinase S6K also regulates SREBP processing downstream of mTORC1 (240). (3) mTORC1 phosphorylates lipin1 and regulates nuclear entry of SREBP mature form through an unknown mechanism (241). While cholesterol plays crucial roles in malignant properties of cancer cells, whether blocking cholesterol biosynthesis by statins, HMGCR inhibitor, reduce the risk of cancer onset or mortality is controversial at present; the statin effects could be dependent on types of cancer (242–244). To plausibly explain this, inhibiting HMGCR increases LDL uptake through upregulation of LDLR expression, which could enable cancer cells to acquire sufficient amounts of cholesterol. LDL levels are much higher in humans than mice. Therefore, inhibiting SREBP could be a more direct target to treat a broad range of cancers.

An aberrant accumulation of CE in lipid droplets via ACAT1 is found in prostate (245) and pancreatic cancer tissues (246). Pharmacological or genetic ACAT1 inhibition attenuates cancer cell proliferation and invasive activity *in vitro* and tumor growth and metastasis in xenograft models (245, 246). Blocking ACAT1 causes an increase in free cholesterol at the ER, which inactivates SREBP-1 but not SREBP-2, reducing cholesterol synthesis and uptake (245). Increased ER free cholesterol levels may induce ER stress and cancer cell apoptosis (246). Furthermore, ACAT inhibition blocks activation of Akt partly though a reduction of cellular arachidonic acid (245), and reduced SREBP activity and cholesterol biosynthesis could also impair the integrity of lipid rafts and suppress Akt activity. Therefore, ACAT1 is an attractive therapeutic target for certain types of cancer where large accumulations of CE are observed.

Cancer immunotherapy targeting PD-1 expressed on T cells or PD-L1 expressed on cancer cells is clinically used. In these therapies, CD8^+^ T cell activity is crucial for successful achievement. Recent work by Yang et al. (247) showed that blocking ACAT1 activates T cells; ACAT blockage increases PM cholesterol levels and enhances T-cell receptor clustering and signaling, which in turn potentiates effector function and proliferation of these cells to suppress tumor growth in mice. Inhibiting ACAT1 further improved anti-tumor efficacy of anti-PD1 antibody (247). Given the additional suppressive effects of ACAT1 inhibitor on malignant properties of cancer cells, ACAT1 blockage thus has dual beneficial effects in cancer therapy. However, proliferation of CD8^+^ T cell depends upon SREBPs and cholesterol biosynthesis (248), creating difficulties in targeting SREBP pathway to combat cancer. Developing specific drug delivery systems could overcome this barrier.

### Oxysterols and cancer

Serum cholesterol levels are positively correlated with serum 27-OHC, the most abundant oxysterol (213). Hypercholesterolemia is a risk factor of breast cancer, and 27-OHC is involved in the pathophysiology of human breast cancer (249). Expression of CYP27A1, which converts cholesterol to 27-OHC is positively correlated to tumor grade in human breast cancer (250). In aggressive tumor tissues, high CYP27A1 expression was found in both tumor cells and tumor-associated macrophages (TAMs) (250). 27-OHC administration increases the growth of human breast cancers, and CYP27A1 inhibition suppresses tumor growth in xenograft models (250, 251). Further, abrogation of Cyp7B1, which catalyzes the conversion of 27-OHC to 7α, 27-di-OHC resulting in 27-OHC accumulation in plasma and tumor tissues, accelerates tumor growth in a mouse estrogen receptor-positive mammary adenocarcinoma model (250). Reduced expression of CYP7B1 is correlated with estrogen receptor-positive breast cancer aggressiveness (251). 27-OHC exerts pro-tumor effects on breast cancer cells by acting as an estrogen receptor agonist (as a SERM). Additionally, 27-OHC promotes metastases of not only estrogen receptor-positive and -negative breast cancer cells but also of melanoma, lung cancer cells, and pancreatic cancer cells (252). The pro-metastatic roles of 27-OHC depend partly on LXR activity of tumor cells (250) and on immune cells within tumor microenvironment (252). 27-OHC affects several types of immune cells, increasing the number of polymorphonuclear-neutrophils and γδ-T cells, and decreasing CD8^+^ T cells (252).

In contrast to breast cancer, CYP27A1 expression is negatively associated with aggressiveness of prostate cancer; patients whose tumors express higher *CYP27A1* mRNA exhibit lower tumor grade and longer disease-free survival (253). 27-OHC suppresses growth of human prostate cancer cells at least partially by inactivating SREBP-2 pathway (253).

### Sterol transporters and cancer

Lipid transport proteins can modulate malignant properties of cancer cells. ABCA1 has an anti-cancer activity dependent on lipid transport activity (254). ABCA1 expression levels are inversely correlated with tumor aggressiveness in prostate cancer. The *ABCA1* gene promotor is hypermethylated in the tissues of prostate cancer, suppressing its expression (255). ABCA1 deficiency causes an increase in mitochondrial cholesterol content, which promotes survival of cancer cells (254).

Studies using LXR agonists showed that activation of LXR reduces cellular cholesterol content in glioblastoma cells through increased cholesterol export and decreased cholesterol uptake, which suppresses the growth of glioblastoma cells *in vitro* and in xenograft models (256, 257). LXR agonists also suppress metastases of melanoma and prolong animal survival, at least partially though LXRβ-dependent upregulation of apoE expression, in several *in vivo* models (258). Furthermore, accumulation of endogenous 4α-monomethylsterols by genetic manipulation leads to LXR activation and inhibits tumorigenesis in a mouse model (259).

Several ABCA family transporters associate with overall survival of serous ovarian cancer patients. Patients with high expression of ABCA1, ABCA6, ABCA8, or ABCA9 in primary tumors exhibit reduced survival (260). Furthermore, combined expression of ABCA1, ABCA5, and either ABCA8 or ABCA9 leads to poorer survival (260). In this cancer type, ABCA1 seems to have an opposite effect on malignancy by unknown mechanisms. The roles of ABCA5, ABCA8, and ABCA9 are not well-understood.

Independently of LXR/RXR-mediated transcription, ABCA1 expression is regulated by cell density in A431 human epidermal carcinoma cells (261). In cells at low density, focal adhesion kinase (FAK) inactivates the transcription factors Foxo3 and TAL1, resulting in suppression of ABCA1 expression. In contrast, at high density, FAK activity is suppressed, and ABCA1 expression is upregulated (261). Cell density-dependent ABCA1 expression regulates PM lipid compositions (261); cells expressing ABCA1 at high levels contain less cholesterol and ganglioside in the PM compared to those expressing ABCA1 at low levels (94, 114, 261). ABCA1 modulates trans-bilayer distribution of cholesterol at the PM (262). Collectively, in addition to a well-known role in HDL biogenesis, ABCA1 acts to fine-tune membrane lipid compositions to modulate PM functionality that may alter cancer cell phenotypes in a cell type-dependent manner.

Macrophage ABCA1 and ABCG1 also play roles in tumor growth. Deficiency in either ABCA1 or ABCG1 in macrophages attenuates tumor growth in xenograft models (263, 264). Deletion of these genes in macrophages converts the tumor-promoting M2 phenotype to the anti-tumor M1 phenotype TAMs within the tumor microenvironment (263, 264).

Additional lipid/sterol transporter is also involved in human cancer. Higher expression of ORP5, an ER membrane anchored ORP, associates with poor prognosis of patients with pancreatic cancer (265). ORP5 expression levels are positively correlated with cell proliferation and invasive activity. Of mechanistic interest, ORP5 interacts with mTOR, and enhances mTORC1 activity (266).

## Concluding remarks

Cells are highly compartmentalized at both the cellular and organelle levels. Cholesterol, oxysterols, and intermediate sterols move dynamically in cells with correct cellular distribution, being paramount to regulating cellular functions. Cellular sterol transport must be spatiotemporally regulated; however, the mechanisms of this are not fully understood. Compared to cholesterol, much less is known about the transport of oxysterols and intermediate sterols. Additionally, elucidating sterol metabolism and transport mechanisms in disease via pathophysiologically relevant models, such as patient-derived iPS- cell systems and organoid culture, could help identify novel therapies for human diseases. State-of-the-art technologies including genome editing, imaging, and multi-omics may provide new insights into how cells handle sterols in physiological and pathophysiological conditions.

Experimental evidence demonstrates that sterols and sterol metabolism play crucial roles in various aspects of human diseases, not only in atherosclerosis and cancer, but also those not reviewed here, including Alzheimer's disease and other neurodegenerative disorders, and infection among others. Sterol metabolism is an attractive target in disease treatment. Several drugs targeting sterol metabolism are clinically used to treat patients currently, including statins to block cholesterol synthesis and enhance LDL uptake, Ezetimibe to block NPC1L1-mediated absorption of dietary cholesterol, and the anti-PCSK9 monoclonal antibodies (Evolocumab and Alirocumab) to block LDLR degradation and reduce plasma LDL. New potential therapeutic target molecules include SREBPs and SREBP regulators, LXR, ABC transporters, ACAT, and sterol hydroxylases, as a growing number of preclinical and clinical studies targeting these molecules are ongoing. Given the importance of target localization, development of novel drug delivery systems to address associated issues will also be key.

## Author contributions

YY designed and wrote the article. MR contributed to writing and revising the article. Both authors approved the final version.

### Conflict of interest statement

The authors declare that the research was conducted in the absence of any commercial or financial relationships that could be construed as a potential conflict of interest.
